# Supplementary material for: Mediterranean Diet, Vitamin D, and Hypercaloric, Hyperproteic Oral Supplements for Treating Sarcopenia in Patients with Heart Failure—A Randomized Clinical Trial
Source: Nutrients. 2023 Dec 28;16(1):110. doi: 10.3390/nu16010110 (PMC10781070; doi:10.3390/nu16010110)
Supplement: Supplementary file 1 [file nutrients-16-00110-s001.zip › nutrients-2760470-supplementary.pdf]

Table S1. Composition for 100 ml of the standard hypercaloric hyperproteic oral supplement.

Characteristics	Standard OS
**Kcal**	141
**Energeric density (Kcal/mL)**	1.4
**Fat** (g)	5.5
Saturated (g)	1.3
Monounsaturated (g)	2.6
Polyunsaturated (g)	1.6
Omega-6 fatty acids (g)	0.96
Omega-3 fatty acids (g)	0.52
EPA+DHA (mg)	385
**Carbohydrates** (g)	14.5
Sugar (g)	0.86
**Fiber** (g)	1.7
**Proteins** (g)	7.4
**Osmolality** (mOsm/L)	255-375
